# Wessex Branch of the Association of Clinical Pathologists, December 1990 Meeting

**Published:** 1991-03

**Authors:** 


### Wessex Branch of the Association of Clinical Pathologists: Winter Meeting

### Held at the Postgraduate Centre of the Royal Devon & Exeter Hospital, Thursday 6 December 1990

### THE DIFFERENTIAL DIAGNOSIS OF LYMPHOMAS AND GERM CELL TUMOURS USING IMMUNOHISTOCHEMISTRY

**M. Sweerts, P. J. Hall, N. Rooney**
Bristol Royal Infirmary

The distinction between mediastinal germ cell tumours and lymphomas can be difficult, especially on small biopsies. In particular, the number of infiltrating lymphocytes in germ cell tumours can complicate the interpretation of Leucocyte Common Antigen staining. There have been reports of B cell markers MB1 and MB2 staining germ cell tumours. This study aimed to assess the reliability of a panel of antibodies in differential diagnosis of lymphomas and germ cell tumours.

Eight antibodies commonly used in lymphoma panels (LCA, L26, MB1, MB2, CD21, UCHL-1, CD3, and CD30), the germ cell tumour marker, placental alkaline phosphatase and EMA were applied to paraffin sections of 16 seminomas, 15 teratomas and one mixed tumour. Reactivity was demonstrated using a streptavidin-biotin peroxidase method. The sections were screened by two investigators for reactivity with the tumour elements, lymphocytic infiltrate and normal testicular tissues. Antibodies MB1, MB2 and BerH2 reacted with seminomas. Occassional staining of undifferentiated teratoma cells with CD3 was observed. Tumour cells were negative with LCA, L26, CD21 and UCHL-1. MB2 (and occasionally MB1 and UCHL-1) stained mature germ cells and Leydig cells. In conclusion staining with MB1, MB2 or UCHL1 should not be regarded as diagnostic of lymphoma. LCA, L26, CD21 and CD5 should be included in any panel used in the diagnosis of mediastial tumours.

### INVASIVE FUNGAL INFECTIONS IN EXETER HAEMATOLOGY PATIENTS

**M. Shields**
Area Department of Pathology, Exeter

Current haematology practice induces prolonged episodes of severe neutropenia putting patients at high risk of developing invasive fungal infections. In addition it is well recognised that outbreaks of such infections are associated with building works. Recent experience in the Exeter Haematology Unit demonstrate the severity of the problem. Between December 1989 and May 1990 twenty patients with a wide variety of haematological diagnoses experienced prolonged episodes of severe neutropoenia. Despite a policy of reverse barrier nursing in cubicles fitted with high efficiency particulate air filtration systems and early use of systemic amphoteracin there were three proven and two suspected fatalities from invasive pulmonary aspergillosis. At this time major excavation work was being carried out as part of the Wonford site rebuild. The Haematology Unit directly overlooks this site. The reported cases emphasise the difficulty in making an early diagnosis of pulmonary fungal infection and the poor response to treatment in established cases. All our severely neutropenic patients are now given oral Itraconazole prophylaxis and the isolation procedures have been revised. We have had no confirmed cases of invasive aspergillosis since May 1990.

### THEATRE OVERSHOES: PART OF INFECTION CONTROL HISTORY?

**H. Humphreys**
Bristol Royal Infirmary

The use of theatre over-shoes is said to reduce floor bacterial counts and hence post-operative sepsis. These are worn by occasional staff or visitors on entering the operating theatre area itself but are inconvenient and contribute to theatre costs. We compared theatre floor bacterial counts during two consecutive two-week periods when over-shoes were and were not worn using contact plates placed at 5 different sites four times a day. The over-shoes did not lead to a significant reduction in total counts. At one site counts were higher with over-shoes. *Clostridium perfringens* was not isolated during either period but *Staphylococcus aureus* was recovered more frequently when over-shoes were worn. Theatre over-shoes do not reduce floor bacterial counts but are expensive and inconvenient and as in Intensive Therapy Units should be dispensed with as an infection control measure.

### ACUTE COMPARTMENT SYNDROME: A RARE COMPLICATION OF THEOPHYLLINE TOXICITY

**O. McDonald Nwose, J. P. Begley**
Bristol Royal Infirmary

Severe theophylline toxicity (therapeutic range 55–110 umol/1; toxic > 193 umol/l) results in seizures, cerebral hypoxia, arrhythmias and cardiorespiratory arrest. We report a case in which these features were complicated by an acute compartment syndrome.

An asthmatic (Male, 23 years) presented 2 hours after taking 70 "Slo-Phyllin" capsules with alcohol. On presentation, a serum theophylline of 70 umol/l was associated with serum concentrations: $Na^+$ 148, $K^+$ 2.4, $Mg^{2+}$ 1.7, $PO_4^{2+}$ 0.64, glucose 12.2 mmol/l and alcohol 149 mg/dl. After initial treatment with Ipecacuanha the serum theophylline rose to a peak of 1210 umol/l 8 hr later. Supraventricular tachycardia, treated with amiodarone was followed by seizures unresponsive to diazepam or midazolam but controlled by thiopoentone.

### A NOVEL DIAGNOSTIC TEST FOR PRIMARY BILIARY CIRRHOSIS

**J. P. Begley, R. E. Berry, R. M. Denton & D. Stansbie**
Bristol Royal Infirmary

Primary biliary cirrhosis is characterised by serum anti-mitochondrial antibodies (AMA) which are directed against components of a family of oxoacid dehydrogenases namely pyruvate (PDH), branched chain 2-oxoglutarate (OGDH) dehydrogenase. We have shown that PBC sera inhibit the catalytic activity of PDH > BCOAD > OGDYH. The aim of this study was to explore the diagnostic potential of PBC sera inhibition of PDH activity. Sera were obtained from normal women (95) and patients with PBC (53), chronic active hepatitis (CAH; 40), progressive systemic sclorsis (PSS; 35), miscellaneous chronic liver disease (CLD; 28) and surgical obstructive jaundice (SOJ; 27). Diagnosis were made on the basis of clinical, immunological and histological grounds. PDH activity (pig heart 1 unit/ml, 2 ul) was measured by a coupled spectrophotometric method with or without the addition of PBC sera (2 ul) immediately before asay. PDH activity (intra assay CV, 4%) was note affected by the presence of sera from controls or SOJ patients. In marked contrast all sera from controls or SOJ patients. In marked contrast all sera from PBC patients showed significant PDH inhibition (greater than 3 × assay CV) as did 3 CAH sera and 3 PSS sera. In this series the diagnosis of PBC by PDH inhibition showed a specificty of 95% and a sensitivity of 100% and has great potential as a diagnostic tool.

### HISTOLOGICAL LESIONS FOLLOWING WIRE INSERTION IN BREAST BIOPSIES: EARLY GIANT CELLS

**L. R. A. Day, J. D. Davies, J. S. Armstrong & M. Priovoulou-Papaevangelou**
Regional Breast Pathology Unit, Southmead Hospital

During the first year of implementing the Avon Breast Screening service, draining the three health districts of Frenchay, Southmead and Bristol and Weston, 120 women were submitted to surgical breast biopsy. In order to facilitate the accurate resection of impalpable mammographic abnormalities hooked wires were inserted into the breast. Three different types of wire were used, viz Mammalok-plus (69 cases), X-Reidy (12 cases) and Nottingham (8 cases). These remain in place for one to six hours before surgery. The histological changes which develop as a consequence of this procedure have not been previously described.

Fifty wire insertion biopsies (41 Mammalok-plus, 6 X-Reidy and 3 Nottingham) and 50 routine biopsies without wires were examined histologically. Changes caused by surgical trauma were excluded by ensuring that all resection margins were marked. Numerous haematoxylin and eosin stained sections from each biopsy were studied. Forty five out of 50(90%) cases with wires showed areas of extravasated erythrocytes, lymphocytes, macrophages and numerous polymorphonuclear leucocytes. Two cases showed giant cells. Eight out of 50 (16%) cases without wires showed minimal extravasation of erythrocytes with only occasional polymorphs and lymphocytes. These latter changes were often associated with the surgical resection line and are thus probably caused by trauma secondary to the surgery. No giant cells were seen.

It is important to be aware of the histological appearance commonly associated with wire insertion to prevent erroneous diagnoses of fat necrosis or infection. Giant cells have previously been found in the circulation and can migrate to areas of inflammation; they are not thought to have formed at the site of wire insertion in one to six hours.

### CONGENITAL DYSERYTHROPOIETIC ANAEMIA

**E. L. Blundell**
Southmead Hospital

A baby girl (full term, normal delivery) admitted to the Special Care Baby Unit, one day after birth was found to have hepatosplenomegaly and to be anaemic (Hb 11.22 g/dl). Numerous nucleated red cells were seen on the film. She was presumed to be infected and made a satisfactory response to intravenous antibiotics and fluids. Subsequent follow up one week later showed a further fall in haemoglobin (Hb 7 g/dl) and she required transfusion. She had an elevated serum bilirubin (65 µmol/L). There deficient in haematinics and had no abnormality of red cell enzymes. Screening for evidence of congenital infection was negative. Bone marrow aspiration showed morphology typical of Type II Congenital Dyserythropoietic Anaemia (C.D.A.II). She has none of the serological abnormalities usually associated with this condition; in particular, Ham's test has been negative on several occasions. Her management is by regular blood transfusion with appropriate iron chelation therapy. Her brother, born 3 years later with a haemoglobin of 6.1 g/dl, shows similar bone marrow morphology and lacks the serological characteristics of C.D.A.II. Since the classification of congenital Dyserythropoietic Anaemia by Heimple and Wendt in 1968, many variant forms have been described. Clinical problems due to iron overload may arise where regular transfusions are given, and aplastic crises due to parvovirus infection have been documented. The cause of C.D.A. is not known. Many workers have demonstrated abnormalities in glycosylation of erythrocyte membrane proteins but it is unclear whether this represents the primary defect or purely reflects the increased rate or red cell maturation.

### MODIFICATION OF PROSTATIC EPITHELIAL SECRETIONS IN GRANULOMATOUS PROSTATITIS

**J. Dhundee, A. G. MacIver**

Southmead Hospital, Bristol

The aim of our study was to examine the inflammatory components, their distribution and their effect on prostatic duct epithelium in granulomatous prostatitis, using an immunohistological technique. Although relatiely uncommon, granulomatous prostatitis is a distinct inflammatory condition and possible causes include infection (Mycobacterial or fungal) and previous prostatic surgery. The morphology of individual granulomata has been likened to that of a rheumatoid nodule, and an autoimmune mechanism has been postulated. In most cases, however, the aetiology is unknown and it may be a reaction to extravasated secretions released from dilated ducts obstructed as a result of nodular hyperplasia. We found that the cellular inflammatory infiltrate in both focal periacinar and diffuse granulomatous prostatitis consisted predominantly of T lymphocyts and macrophages but few B lymphocytes occurred. Fibrinogen-related antigen was not detected in the granulomata. In contrast a small amount was present in focal prostatic infarcts. There was significant reduction in prostatic specific antigen and prostatic acid phosphatase immunoreactivity of ductal epithelium in areas of granulomatous inflammation whereas these products were detectable in areas of infarction. This suggests that T lymphocyte or macrophage-derived, cytokine-mediated alteration to epithelial secretion may be taking place in granulomatous prostatitis.

### CHANGES IN ANTIBIOTIC SENSITIVITY OF URINE AND SYSTEMIC ISOLATES IN A DISTRICT GENERAL HOSPITAL 1984–1990

**N. M. Brown**
Southmead Hospital, Bristol

Sensitivity test results of all significant isolates cultured at Southmead Hospital, Bristol have been recorded since 1984. They have divided into urine and systemic (all non-urine) isolates. Results were also divided by site of the origin into hospital or community. Sensitivity testing was done using a modified Stokes' method. This method has not changed since 1984. Urine coliform and *Proteus* spp. from the community have shown no great change in sensitivity to antibiotics commonly used to treat urinary tract infection, except that sensitivity of *Proteus* spp. to trimethoprim fell from 60% to 40% in 1986 and 1987. This increased resistance cannot be explained easily. Sensitivity of hospital urine isolates was slightly lower than those from the community, as would be expected, but has not changed since 1984.

Interestingly, urine *Pseudomonas* spp. isolates from hospital and community have shown a fall in sensitivity to ciprofloxacin from 99% and 100% to 94% and 92%, respectively. Meanwhile sensitivity to carbenicillin for community isolates has risen from 77% to 94%. These changes may reflect changes in treatment of peudomonas urinary tract infection since the introduction of ciprofloxacin. With systemic isolates the significant change has been a fall in sensitivity of hospital coliform isoplates to cefuroxime from 85% in 1984 to 71% in 1988. There has also been a fall in sensitivity to ampicillin from 57% to 45%. This is important as cefurime is widely used in this hospital for treatment and prophylaxis. Again these changes may reflect antibiotic usage and were not seen in community isolates.

10% of *Haemophilus influenzae* isolates were resistant to ampicillin. There has been no change since 1984. This is consistant with the level seen in the South of England. Overall antibiotic sensitivity appears to have changed little since 1984. However, if the trends described continue they will have major implications for design of regimens for prophylactic and blind antibiotic therapy.

### FAECAL CONCRETIONS IN THE APPENDIX

**B. F. Warren, H. S. Rigby, D. Ball, J. Begley, J. W. B. Bradfield**
Bristol Royal Infirmary

The role of faecoliths in the cause of acute appendicitis has remained under debate for most of this century. There has been a probable change in incidence of faecoliths over the years. Boaers in 1939 reported 67% of resected appendices to contain faecoliths whereas more recent studies have indicated a much lower figure. All these studies have failed to agree on the definition or classification of faecoliths which might explain some but not all of the variation. We studied 200 surgically resected appendices. A faecolith was defined as incompressible and partly calcified. They were further classified according to Lloyd-Davies and Forbes by their radiogical appearance. Our overall incidence was 11.5%. There was no difference in incidence of faecoliths between appendices with or without appendicitis. We saw a greater incidence of more heavily calcified faecoliths in the elderly. We believe there has been a real change in the incidence of faecoliths over the last 50 years, which may be due to diet or environment.

### AN UNUSUAL SEPTICAEMIA

**Marina Morgan**
Public Health Laboratory, Heavitree, Exeter

Five days after a trivial dog-bite, a 37 year old asplenic engineer was transferred for Intensive Care. He was fitting and had D.I.C. and ARDS, and incipient gangrene of the tip of his nose. He had initially presented 2 days previously with septic shock and malar purpura. With a peripheral blood film showing Gram-negative, intracellular rods, ciprofloxacin and penicillin therapy was instituted for a presumptive diagnosis of DF-2 (Dysgonic fermenter type 2) septicaemia. This diagnosis was finally confirmed eleven days after admission when the organism was grown from blood cultures taken on day one. Despite all measures, he remained deeply unconscious with an abnormally slow EEG but normal CT scans and CSF examination. He was considered to have irreversible cortical damage. The decision was made to withdraw active treatment if no response occurred within the next few days. After 22 days of intensive care, a flicker of movement was observed in one arm. The next day he opened his eyes, and thereafter his CNS function dramatically improved. Discharged home 52 days after admission, with no congnitive impairment and only a mild residual foot drop, he remains well and is continuing penicillin prophylaxis. DF-2 (Capnocytophaga canimorsus), is a commensal of the oral flora of dogs and cats, causing a fulminant septicaemia in asplenic/cirrhotic patients with 50% mortality. The recovery of this patient from such a prolonged state of apparent brain failure is salutory from the point of view of clinical management. Finally, prophylactic penicillin post splenectomy would undoubtedly have prevented this septicaemia.

### THE NATURAL HISTORY OF MID/MODERATE DYSKARYOSIS–IMPLICATION FOR THE SREENING PROGRAMME FOR CERVICAL CANCER

**M. Jenkins**
Bristol Royal Infirmary

The most appropriate management of women with mild and moderate dyskaryosis is controversial, largely because the natural history of such abnormalities is unknown. I have followed 735 women with mild and moderate dyskaryosis who were managed by cytological surveillance, over an 11 year period. The smears from these cases plus those from an equal number of age-matched normal controls were reviewed. The eventual outcome of these abnormalites presented and the prognostic significance of various smear features (grade and distribution of dyskaryosis, presence of endocervical cells, viral changes, blood and inflammatory cells) discussed. The results show that cytological surveillance for mild/modernate dyskaryosis is safe and suggest possible reasons why some lesions progress and others regress.

### SHORT COURSE ANTIMICROBIAL THERAPY OF INTRACRANIAL ABSCESS

**B. Kirkpatrick**
Frenchay Hospital, Bristol

It is standard practice for patients with intracranial abscess to receive antibiotics empirically for 6 weeks or longer. We suspected that the administration of these agents was unnecessarily prolonged and now base the decision to stop treatment on the patient's clinical response, on the fall of the serum C reactive protein to normal, and on serial CT scans. To date, 14 patients, 8 with brain abscess and with subdural empyema have been treated using these criteria. The choice of antibiotics was determined by the susceptibility of the isolates obtained at initial surgery. Eleven patients were treated for 16 days or less (mean 13.9) and follow up ranged from 1–22 months (mean 11.5), during which time there were no relapses. The remaining patients whose abscesses were otogenic received more prolonged courses (26 days, 30 days and 10 weeks). In these patients the intracranial sepsis appeared to have been eradicated at an early stage, but therapy was extended either because the CRP was persistently elevated or it rose after an initial decline. This was subsequently atributed to an undiagnosed deep vein thrombosis (1 patient), pulmonary emboli (1 patient) and failure to treat adequately the underlying aural pathology (1 patient).

These results suggest that patients with intracranial sepsis may be successfully and adequately treated with much shorter courses of antibiotics than are currently recommended, thereby significantly reducing hospital stay, costs, and the incidence of side-effects associated with the therapy.

Despite the memorably foggy conditions in the early morning on the motorway, about 40 members attended this meeting, under the Presidency of Dr J. D. Davies (Bristol). A determined effort is made to include papers from all disciplines of clinical pathology, and to indicate that they are especially welcome if their appeal crosses the boundaries between disciplines. Thirteen papers were presented by trainee members at the three scientific Sessions after the Business meeting in the morning. The preliminary organisation for this whole-day meeting was carried out by Dr Elizabeth Mackenzie (Southmead), in conjunction with our hosts in Exeter. The President, Dr Ian Cruickshank and Professor P. P. Anthony (Exeter) chaired the Scientific Sessions.

